# Designing user-centered evaluations: Leveraging beta-testing results to develop process evaluation interview questions for the AMPLIFY program

**DOI:** 10.1371/journal.pdig.0001477

**Published:** 2026-07-07

**Authors:** Jami L. Anderson, Laura Q. Rogers, Wendy Demark-Wahnefried, Michelle Y. Martin, Maria Pisu, Yu-Mei Schoenberger-Godwin, Dorothy W. Pekmezi, Ivan I. Herbey, Kevin R. Fontaine, Kelly Kenzik, Robert A. Oster, Larry Hearld, Tapan Mehta, John Waterbor, Nataliya V. Ivankova

**Affiliations:** 1 Department of Family and Community Medicine, Heersink School of Medicine, University of Alabama at Birmingham, Birmingham, Alabama, United States of America; 2 Division of General Internal Medicine and Population Science, Department of Medicine, Heersink School of Medicine, University of Alabama at Birmingham, Birmingham, Alabama, United States of America; 3 O’Neal Comprehensive Cancer Center, University of Alabama at Birmingham, Birmingham, Alabama, United States of America; 4 Department of Clinical and Diagnostic Sciences, School of Health Professions, University of Alabama at Birmingham, Birmingham, Alabama, United States of America; 5 Department of Preventive Medicine, University of Tennessee Health Science Center, Memphis, Tennessee, United States of America; 6 Department of Health Behavior, School of Public Health, University of Alabama at Birmingham, Birmingham, Alabama, United States of America; 7 Department of Surgery, School of Medicine, University of Alabama at Birmingham, Birmingham, Alabama, United States of America; 8 Department of Surgery, Brigham and Women’s Hospital, Harvard University, Boston, Massachusetts, United States of America; 9 Department of Health Services Administration, School of Health Professions, University of Alabama at Birmingham, Birmingham, Alabama, United States of America; 10 Department of Epidemiology, School of Public Health, University of Alabama at Birmingham, Birmingham, Alabama, United States of America; University of Vic - Central University of Catalonia, SPAIN

## Abstract

Refining digital health programs with end-user feedback is essential throughout program lifecycles to ensure user-centered perspectives are incorporated during all program development. Results from beta-testing “think aloud” interviews exploring program engagement, satisfaction, motivation, and usefulness may be leveraged to support development of interview questions for a subsequent user-centered process evaluation implemented during program efficacy testing. We describe how results from beta-testing “think aloud” interviews were utilized to inform the design of user-centered process evaluation interview questions for implementation during program efficacy testing of AMPLIFY, a web-based diet and exercise program for older cancer survivors. Beta-testers (n = 15) were asked to complete three “think aloud” interviews via a videoconference platform over a 6-month period; 40 interviews were completed, accounting for early dropouts. Transcripts were analyzed using a multi-step process: 1) suggested refinements from beta-testing “think aloud” interview results were catalogued and organized according to whether the program feature was liked, disliked, or confusing to the beta-tester; and 2) suggestions for refinements were categorized based on feasibility to adopt (feasible/not feasible), the type of recommended change (formatting/content), and underlying improvement goal (engagement, satisfaction, motivation, usefulness). Refinements considered feasible to adopt were categorized into formatting refinements (changes to the presentation/organization of the program) and content refinements (additions, deletions, or changes to the information provided in the program). Refinements were also categorized by relationship to four process evaluation constructs: engagement, satisfaction, motivation, and usefulness. Results informed user-centered process evaluation interview questions for implementation during program efficacy testing; such that, subsequent semi-structured interview questions were developed based on these evaluation constructs and underlying improvement goals. The application of beta-testing results to refinements and process evaluation provide a potential guide for other researchers developing and evaluating digital health interventions.

## Author summary

Beta-testing is useful to refine and tailor programs based on feedback from participant interviews. Results from beta-testing can be used during other stages in the program lifecycle to ensure participant feedback is retained throughout the refinement, testing, and evaluation stages. However, few approaches have been described to link beta-testing results with subsequent process evaluations. We describe how beta-testing participant interview results were used to support the development of user-centered process evaluation interview questions for implementation during testing of the AMPLIFY program. AMPLIFY beta-testers (n = 15) were asked to complete three “think aloud” interviews via a videoconference platform over a 6-month period. Transcripts were analyzed using a multi-step process: 1) suggested refinements from beta-testing “think aloud” interview results were organized according to whether the program feature was liked, disliked, or confusing to the beta-tester; 2) suggestions for refinements were then categorized based on feasibility to adopt, type of recommended change, and improvement goal; and 3) refinements considered feasible to adopt were categorized into formatting refinements and content refinements. We then categorized refinements by four process evaluation constructs: engagement, satisfaction, motivation, and usefulness. We used these results to inform user-centered process evaluation interview questions for implementation during program efficacy testing.

## Introduction

**Fig 1:**
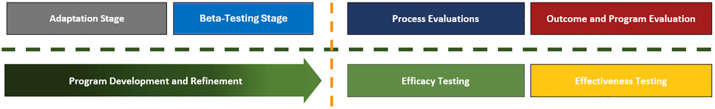
Basic lifecycle of a digital health program [3].

Beta-testing is a critical step in digital health program refinement and is typically conducted prior to programmatic efficacy or effectiveness testing [1,2]. Beta-testing often incorporates rigorous qualitative components, such as “think aloud” interviews, exploring perspectives of end-users and incorporating their feedback into the program during the refinement process [1,2] (Fig 1). “Think aloud” interviews combine usability tasks with follow-up semi-structured interviews and are increasingly used to inform and refine features of digital health programs during the beta-testing stage [1,2,4]. Refinements suggested during beta-testing “think aloud” interviews, and subsequently incorporated into a web-based program, ensure the program maintains a user-centered focus and provide valuable insights into underlying program improvement goals [1,2,5]. While beta-testing web-based programs with “think aloud” interviews is increasingly common [2,6], few resources are available to assist in leveraging beta-testing data to inform the design of subsequent user-centered process evaluation interviews conducted during program efficacy testing (Fig 1) [3]. Fig 1 represents a common lifecycle of digital program development including multiple stages of adaptation and refinement followed by program testing and evaluation.

Interviews with study participants are important components of user-centered process evaluations to incorporate user feedback and maintain a user-centered perspective throughout the evaluation [7–10]. In particular, results from beta-testing “think aloud” interviews may be useful to identify underlying program improvement goals; providing insights into evaluation constructs related to participant engagement, satisfaction, motivation, and perceived usefulness of a web-based program. Linking beta-testing “think aloud” interview results to the development of a subsequent process evaluation interview guide may: 1) ensure a user-centered focus is maintained throughout the study timeline, including the process evaluation conducted during program efficacy testing; and 2) ensure all concerns and aspects of the program highlighted by beta-testers are subsequently found acceptable and useful in the larger study sample recruited for program efficacy testing (Fig 1). Therefore, the aim of this article is to describe an approach to linking beta-testing “think aloud” interview results with the development of subsequent process evaluation interview questions to ensure a user-centered perspective is maintained throughout the development timeline for a web-based diet and exercise program, AMPLIFY. The approach involved first conducting qualitative content analysis of “think aloud” interviews followed by categorization and linkage of qualitative results to evaluation constructs appropriate for a process evaluation.

### AMPLIFY program

AMPLIFY (AiM, Plan, and act on LIFestYles) is a web-based diet and exercise program developed by adapting efficacious diet and exercise interventions into a web-based platform [7,8]. AMPLIFY is designed to support cancer survivors achieve and maintain a healthy Body Mass Index (BMI) and consistent exercise to prevent the recurrence and secondary onset of cancer [7,8]. The AMPLIFY development and testing timeline included three stages: 1) development and adaptation; 2) beta-testing and refinement; 3) efficacy testing and process evaluation (Fig 1). During AMPLIFY program development and adaptation, two efficacious interventions, BEAT Cancer (a site-based physical activity behavior change intervention) and RENEW (a home-based diet and exercise intervention delivered via mailed print and telephone counseling) [9,10], were adapted for web-based delivery [7,8]. The AMPLIFY program website design was also informed by SurvivorSHINE (the web-based prototype of RENEW, a home-based diet and exercise intervention for cancer survivors) [11]. Three interventions were developed: 1) a 6-month diet and weight loss intervention, 2) a 6-month exercise intervention, and 3) a 12-month combined diet and exercise intervention. Each intervention contained specific features such as informational sessions, daily diet tips, or exercise tracking [7,8]. The diet intervention provided information regarding diet quality, portion-control, meal planning and weight loss specifically tailored for older cancer survivors. The exercise intervention provided information regarding types, duration, and intensity of physical activity recommended for older cancer survivors. The combined intervention incorporated both diet and exercise components into a single program, delivering diet and exercise content simultaneously. All interventions included content on behavior change, such as incremental goal setting, self-monitoring, reinforcement/rewards, and social support. Greater details about the design of the AMPLIFY program are reported elsewhere [8].

## Methods

### AMPLIFY beta-testing procedures

The three AMPLIFY interventions (diet, exercise, and diet/exercise combined) were beta-tested with cancer survivors who were recruited using a centralized process involving two sites (University of Alabama at Birmingham and the University of Tennessee Health Science Center) [8]. Because the AMPLIFY program content was tailored for middle-aged and older cancer survivors ≥ 50 years old, recruitment criteria for beta-testers included being a cancer survivor of an obesity-related cancer, within an age group ≥ 50 years old, residence in an area with wireless internet coverage, and other characteristics [9]. A stratified sampling approach was utilized and focused on generalizable characteristics of race, sex, and rural/urban residence. Beta-testers who met the inclusion criteria were randomly assigned to one of the three interventions (diet, exercise, or combined) and spent 7–12 months testing their assigned intervention.

Ethical approval for the AMPLIFY study, beta-testing, and “think aloud” interviews was obtained from the Institutional Review Board of the University of Alabama at Birmingham. Signed informed consent was obtained before beta-testing activities were initiated and verbal consent was confirmed prior to conducting and recording “think aloud” interviews.

#### Data collection.

Beta-testing “think aloud” interviews were implemented in three phases, based on the length of time the beta-testers were using the website. Phase 1 interviews were conducted after beta-testers spent 1 month using the AMPLIFY website. Phase 2 interviews were conducted after beta-testers spent 2–6 months using the website. Phase 3 interviews were conducted after beta-testers spent 7–12 months using the website. Individual “think aloud” interviews with beta-testers lasted 45–90 minutes and were conducted using a web-based Zoom videoconference platform. Due to rolling enrollment and rapid integration of feedback into program design, a beta-tester enrolled later in the refinement stage would be reviewing results of input provided by prior beta testers.

“Think aloud” interviews consisted of two parts (usability tasks immediately followed by clarifying semi-structured interviews) during which beta-testers were asked to use the website and discuss potential refinements (e.g., elements they liked, did not like, and found confusing). Example usability tasks and follow-up clarifying interview questions are presented in Table 1.

**Table 1:** Example usability tasks and follow-up clarifying interview questions.

	Usability Tasks	Follow-Up Clarifying Interviews
Phase 1 (after 1 month of website use)	Find where you can change your password. Tell me what you are looking at and what you are thinking.	What are some things you don’t like about the website?
	Find your goal for the week. What is your goal?	What do you like about how goal setting is done?
Phase 2 (after 2–6 months of website use)	Go to the support page. Tell me what you are looking at and what you are thinking.	What do you like about the support page of the website?
	Go to Facebook and go to the AMPLIFY discussion group. Tell me what you are looking at and what you are thinking.	What would you like to change about the AMPLIFY Facebook discussion group?
Phase 3 (after 7–12 months of website use)	Go the tools page. Find a tool and tell me what you are looking at, what you are thinking, and why you chose this tool.	What would you like to change about the tools page?
	Find where you enter your tracking data and show me how you would enter it.	What don’t you like about how tracking of your healthy eating or exercise is done?

Questions pertaining to features of central importance to the program and features that changed after incorporating refinements were repeated in each phase of the “think aloud” interviews (e.g., data tracking, goal setting, and informational sessions). To explore supportive program features and ensure all features were tested at least once during the beta-testing process, each phase included several questions unique to that phase only (e.g., questions about daily tips, news, and Facebook).

#### Data analysis.

“Think aloud” interviews were transcribed, verified for accuracy, and analyzed using a 2-step approach: 1) qualitative content analysis and 2) categorization and linkage to evaluation constructs: engagement, satisfaction, motivation, and usefulness.

#### Step 1: Qualitative analysis.

Interview transcripts were coded by two qualitative researchers (IH and JA) utilizing NVivo 12 Plus software (Lumivero, version 12 Plus) with a target of 90% or above for inter-coder agreement [12]. Coding results were regularly reviewed by a qualitative methodological expert (NI) and discussed during research team meetings. A qualitative content analysis approach [13], whereby excerpts of transcribed interviews were categorized based on the content of the excerpt without additional interpretation from the researcher, was utilized to identify programmatic elements beta-testers liked, did not like, or found confusing. For example, beta-testers stated they “liked the My Progress tool within the AMPLIFY website.” Thus, this excerpt was coded under ‘Refinements Participants Liked’ in our coding scheme. Refinements were catalogued according to beta-testing phase (i.e., phase 1, phase 2, or phase 3) and intervention type (i.e., diet, exercise, or combined). All suggested refinements were regularly shared with the AMPLIFY program development team and the team decided the course of action most likely to preserve program fidelity while responding to participant suggestions; including decisions regarding feasibility of adopting the refinement, determination of the type of refinement, expertise required to implement a formatting or content refinement (i.e., AMPLIFY programmatic diet and exercise content expertise or digital program technological expertise), or a determination not to adopt the refinement.

#### Step 2: Categorization and linkage to evaluation constructs.

After beta-testing was complete and determinations made from the AMPLIFY team, refinements were subjected to a secondary categorization and linked to evaluation constructs previously identified by our team from a systematic literature review and conceptual framework on process evaluations conducted during (or just after) efficacy testing of web-based diet or exercise programs involving middle-aged and older adults with chronic disease [14]. This systematic review encompassed multiple databases (i.e., PubMed, Embase, Business Source Premier, and ABI/Inform) to reflect an in-depth exploration of the existing literature most closely aligned with our research [15]. Results from this systematic review and conceptual framework, including justification for the systematic review research question and organization of the conceptual framework, are reported elsewhere [15].

While multiple constructs may influence participants’ willingness to use digital health tools and change their diet and exercise behaviors, results from the systematic review suggested that acceptability and use of web-based programs among older cancer surivors interested in diet and exercise practices may be most influenced by the intersection of participants’ engagement with a program, satisfaction, motivation and perceived usefulness [15]. These four constructs were chosen and organized into a conceptual framework for categorizing AMPLIFY program refinements and to inform development of a semi-structured interview guide of AMPLIFY process evaluation questions [15]. Participant engagement was defined as adherence to the program requirements regarding logins, milestone achievements, and engagement with online program elements (e.g., program website, pages, program content, and program tools) [15–18]. For the purposes of AMPLIFY’s beta-testing, participant engagement was defined and explored both quantitatively (i.e., via AMPLIFY website logins and click depths) and qualitatively (i.e., via “think aloud” interviews). Satisfaction was defined with respect to the degree of alignment between participants’ pre-existing expectations and actual programmatic content [19]. Motivation was defined according the program’s influence on changes to participants’ reported willingness to adopt recommended diet and exercise behaviors [15,20]. Lastly, usefulness was defined in relation to the participants’ perceptions of the value of the program to support improvements in their diet and exercise behaviors [18,20,21].

**Fig 2:**
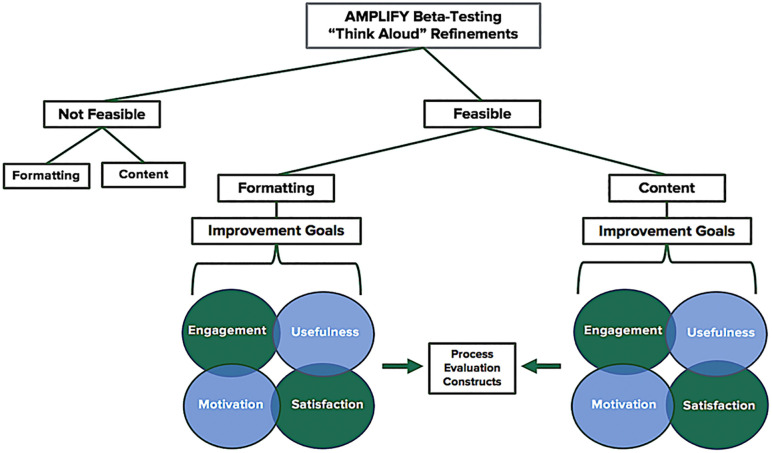
Categorizing refinements identified during “think aloud” interviews.

All refinements suggested by beta-testers were categorized according to: 1) feasibility to adopt suggested changes (i.e., feasible or not feasible); 2) type of recommended change (i.e., formatting or content); and 3) underlying program improvement goals (i.e., increasing program engagement and participants’ perceived satisfaction, motivation, and usefulness) (Table 2). Table 2 provides example illustrative quotes of participants’ suggested refinements and phase of the suggested refinements. In addition, multiple examples of evaluation questions linked to these refinements are found in Table 2. Feasibility was determined by the program development team and scope of the study grant. The refinements were further linked, or aligned, with the four identified process evaluation constructs, as shown in Fig 2. This linkage involved considering each suggested refinement and determining the underlying value and purpose of incorporating the suggested refinement into the AMPLIFY program, based on the definitions of the four process evaluation constructs. Leveraging these four constructs as a framework to program refinement ensured program fidelity and a comprehensive process evaluation reflecting results from our systematic review. Feasible refinements were used to inform the content and organization of semi-structured process evaluation interview questions. These interview questions were developed for implementation during the AMPLIFY program efficacy testing phase. Conducting individual “think aloud” interviews, rather than focus groups, provided the best opportunity for in-depth discussions and testing of the AMPLIFY program to ensure perspectives from each beta-tester was recorded.

**Table 2:** Examples of feasible refinements connected to process evaluation constructs and interview questions.

Beta-Testing Interview Phase	Examples of Refinement (Applicable AMPLIFY Interventions)	Illustrative Quote	Improvement Goal and Connection to Process Evaluation Construct(s)	Examples of Process Evaluation Interview Questions
Formatting Refinements				
Phase 1	Incorporating back arrow to navigate away from a video (All)	“If I were doing the website, when you get through the video session, and you’ve got the green arrow, and you click the green arrow to close that, you got three choices…you can get more information…but it won’t just back automatically. I would probably have a back arrow, going back. That’s the way most websites work, very few websites work where you gotta ‘X’ out of that to get back to the next information block. Most websites have a backwards arrow.”	Improve program usefulness and satisfaction by allowing participants to more easily navigate away from the video session.	• How easily could you navigate the AMPLIFY website? • What features made navigating the website easy/difficult?
	Using dropdown lists of weekly goals vs. filling in a blank (All)	“You just watched a motivational session, and then you can set a goal. With that goal setting, if there’s like a list of goals, other than you fill in the blank, what you want to be your goal.”	Enhance program usefulness and engagement by supporting autonomy for the participant in their progress with the program.	• How did you use the AMPLIFY program to help you reach your goals?
	Providing the way to record weight in decimal points vs. round numbers (Diet and Combined)	“The scales will weigh into tenths, but it seems to me that when I put in a 10th it doesn’t save the 10th. So, if I’m 210.8 versus 210.0, well that was really almost a pound loss but it’s still 210.”	Enhance program usefulness, satisfaction, and motivation to continue with the program by more accurately reflecting participants’ true weight.	• What features did you find useful? • How would you describe your satisfaction with the weight tracking feature?
Phase 2	Incorporating a button to navigate out of the tracking feature of the website (All)	“Now, I take my mouse and I move it. When I move it down, I take the wheel [of the mouse] and when I roll it down, it changes the number. So, the way you will have to avoid that…hit the tab key. You have to tab it down to get out of that box.”	Enhance program engagement and usefulness across different devices (e.g., using a desktop computer and mouse).	• How easily could you navigate the AMPLIFY website? • What features made navigating the website easy/difficult?
	Optimizing the program features across technological platforms (All)	“I type it in, you know, if you hit back, the pounds…you can kind of see it. But, at the end when I hit submit then it had disappeared [on the phone screen]. But, it disappears anyways. I’m hoping that they can still see it because I cannot go and reenter all the weights.”	Enhance program engagement and usefulness by minimizing barriers to utilizing program features.	• What types of devices were you using to access the website? • What did you not like about using the AMPLIFY website on a tablet/smartphone or desktop computer?
Phase 3	Reevaluating or removing acronyms utilized for tools throughout the program (All)	“So, AMPLIFY A.I.M., I thought that was really dumb, using those words [A.I.M.], I don’t like stuff like that. It was just getting through the acronyms.”	Enhance program usefulness and satisfaction by clarifying the purpose of the program tools.	• What features did you find useful in the AMPLIFY program? • What tools did you use most frequently?
Content Refinements				
Phase 1	Providing explanation of the levels of exercise (mild, moderate, strenuous) (Exercise)	“I felt there was a lot of logical information, but maybe they needed to throw in some of that, some of that emotion in there. Kind of give them a little bit of motivation to stay with it.”	Enhance motivation, usefulness and increase engagement with the program by adding more information to program components.	• How easily could you find information about the AMPLIFY program? • How satisfied were you with the amount of information provided in the AMPLIFY program?
	Having a forum for participants to share experiences and success storis (All)	“I would like more participants to share their history with it, that would be good. And maybe just, like a separate, not session, but place that you can go to and read such and such story with this, or something like that. That would be good.”	Enhance motivation, satisfaction, and engagement with the program by creating a private Facebook group.	• How would you describe your satisfaction with the support offered through the AMPLIFY Facebook group?
	Incorporating greater ethnic diversity in the program images (All)	“Initially, when I was looking at the exercise, I did not see things that related to ethnicity. It was just exercise. And, it might be that I was looking more at the illustrations as opposed to the information. That was initially.”	Enhance program satisfaction and motivation by increasing diversity focus.	• How satisfied are you with the AMPLIFY intervention at this point?
Phase 2	Introducing a progress plan or roadmap for the program (All)	“Maybe when you go to the website you can kind of get like a comprehensive overview of the entire one-year program. Like, ‘you know we want to start you out. This is kind of a base level here. The first four weeks or six weeks and then in week seven, eight, and nine we’re going to start getting more goal oriented or more focused.’ This could be a little more advanced or because it was a good progression.”	Increase motivation to remain in the program by clarifying program goals and related milestones.	• How would you describe the purpose of the AMPLIFY program? • What features did you find motivating and why?
	Providing previews of upcoming sessions (All)	“If there’s an interesting topic that’s gonna be on there a certain day and you know that ahead of time, and then you get an alert about, ‘Don’t forget, on Wednesday, there’s going to be a session on this or that.’ Well, you’re likely to listen to it then.”	Enhance program satisfaction and engagement by clarifying expectations and managing participation by including.	• What were you expecting when you joined the AMPLIFY program? • How well did we meet your expectations?
	Decrease the amount of information to recorded daily/weekly (Diet)	“I was surprised about that [amount of information to record]….a little overwhelmed by all the activities and food, especially with the food.”	Improve program engagement and usefulness by minimizing barriers to participating in the program and to utilizing program features.	• What could we change to encourage you to use the AMPLIFY program regularly?
Phase 3	Provide alternative exercises for physical limitations (Exercise)	“Everything was good, you know. But, some of them, I know I couldn’t use because I have problems. Back problems and stuff and a lot of stuff I can’t use. So, I have to improvise and do stuff that would not hurt me.”	Enhance program engagement, motivation, and usefulness by minimizing barriers to physical activity and increasing program participation.	• Now that you have completed the exercise module, how useful did you find the AMPLIFY intervention?
	Continue providing tips for healthy eating at social events (Diet)	“How to attend social events and maintain a healthy diet. It ran through some different ways… Some very specific things about socializing and entertaining.”	Enhance program usefulness and satisfaction by providing relevant information.	• How satisfied were you with the amount and quality of the information provided in the AMPLIFY intervention?
	Include statements about the purpose of repetitive information (All)	“Acknowledge there’s repetition in here and say, ‘okay, we’re going to tell you and we’re going to go over this again because it is real important. We know that the more we can tell you the better it will stick with you. So, we recognize [the repetition]. Because, I thought, didn’t I just do this? And, so be real clear about that.”	Enhance program engagement and motivation by emphasizing important content.	• How did the AMPLIFY program motivate you to improve your diet/exercise?

## Results

AMPLIFY beta-testers included older cancer survivors (mean age 64 ± 10 years) of which 53% were women and 41% were African American with varying residency (rural/urban) in the United States. Seventeen individuals consented to participate in beta-testing; 15 beta-testers began the beta-testing process; one beta-tester missed the Phase 1 interview but completed Phase 2 and 3 interviews; one beta-tester withdrew after completing the Phase 1 interview; and two beta-testers withdrew after completing the Phase 2 interview. Of the two beta-testers who withdrew, one stopped responding to our communication efforts after at least 3 attempts by our team to reconnect and one reported that the program time commitments exceeded their expectations. Considering the length of time beta-testers spent with the AMPLIFY program, the team determined that the remaining 15 beta-testers was sufficient to ensure a comprehensive review of the program. A total of 40 beta-testing “think aloud” interviews were completed from 15 beta-testers and were utilized in this analysis (Table 3).

**Table 3:** Number of beta-testing interveiws by phase.

Phase	Number of Beta-Testers	Number of Interviews
1	15	14
2	14	14
3	12	12

Of 1081 comments made by beta-testers during the three phases of “think aloud” interviews (including all aspects beta-testers liked, disliked, or suggested changing), 550 (50.87%) were comments indicating features participants “liked” and 531 (49.12%) were comments indicating features participants “disliked” or suggested changing. During beta-testing interviews, participants were prompted to explain why they liked or disliked a feature and any suggested changes they recommended. Any statement that a beta-tester provided during an interview that referred to a feature of the AMPLIFY program, was considered a distinct comment. Of the features participants disliked or recommended changing, approximately 94.4% (n = 501) were considered by the program development team as feasible changes or modifications (i.e., within the technological and budgetary capabilities of the program). Refinements considered not feasible to adopt by the AMPLIFY program team included, for example, embedding a calorie counting application or a pedometer into the AMPLIFY website. These types of refinements would require large technical changes to the program but were simple to address with other external (non-AMPLIFY affiliated) pre-existing web-based calorie counting applications or simple pedometers. Fewer features beta-testers disliked or suggested changing were identified in Phase 3 (112 refinements) than in Phase 2 (144 refinements) or Phase 1 (245 refinements). Notably, the number of suggested refinements decreased over the timeframe of the AMPLIFY beta-testing phase as suggested refinements were addressed by the research team in a timely manner as they were described by beta-testers.

Among feasible refinements, 140 (27.9%) were related to formatting and 353 (70.5%) to content. Feasible refinements were then mapped to the four evaluation constructs identified in the literature as important for acceptability and use (engagement, satisfaction, motivation, and usefulness). Table 2 provides examples of feasible refinements supported by quotes from “think aloud” interviews and organized by beta-testing interview phase, underlying improvement goals and process evaluation constructs associated with improvement goals. Examples of corresponding process evaluation interview questions informed by beta-testing data are presented in the last column of Table 2.

### Formatting refinements

Formatting refinements were defined as changes to the presentation or organization of the program. Underlying program improvement goals associated with formatting refinements were linked to at least one process evaluation construct. Notably, the majority of refinements suggested during the Phase 1 beta-testing “think aloud” interviews focused on enhancing the usefulness of the program, ensuring the participants were satisfied with the program, and increasing engagement with the program website.

Alternatively, during Phase 2 interviews, suggested formatting refinements tended to focus on enhancing program engagement and usefulness across different devices by minimizing barriers to utilizing program features. Based on the suggested refinements, the AMPLIFY program was optimized for use across technological devices (i.e., cell phones, computers, and tablets).

Formatting refinements suggested during Phase 3 beta-testing “think aloud” interviews focused primarily on enhancing clarity and usability of the program tools to improve usefulness and satisfaction with the AMPLIFY program.

### Content refinements

Content-related refinements were defined as additions, deletions, or changes to the information provided in the program. These refinements were carefully monitored by AMPLIFY program leadership, including a registered dietician, nutrition scientists, physicians, public health practioners, methodologists, cancer researchers, and a geriatrician to ensure that fundamental program elements were retained and content formats did not alter the diet and exercise recommendations presented within the program. Similar to feasible formatting refinements, underlying improvement goals of content refinements were linked to at least one process evaluation construct. During Phase 1 interviews, beta-testers’ comments were mostly related to different ways of enhancing cancer survivors’ motivation, satisfaction and engagement with the program, e.g., adding more information, incorporating more ethnic diversity and having a forum to share experiences and successful stories. Addressing the latter recommendation, the program development team introduced the AMPLIFY Facebook page to provide an opportunity for program participants to interact with each other, thus enhancing their engagement with the program.

During Phase 2 beta-testing interviews, participants’ feedback continued to focus on program satisfaction, engagement, and motivation; however, the comments were more specific and targeted improvements across the program features, such as clarifying the program goals and expectations, including previews of upcoming sessions, and reducing the amount of self-monitoring by daily and weekly recorded information (i.e., tracking daily steps in the My Progress page). These refinements were further tested with Phase 3 participants.

Phase 3 content refinements mostly focused on program engagement and motivation, but also emphasized program usefulness with the purpose of increasing participation and minimizing barriers to adopting healthy behaviors, e.g., including more exercise routines that are accessible for different physical limitations of older populations, providing skill training related to healthy eating at social events and when dining out, and explaining the purpose of repeated information. Repeating useful information in different contexts within the program was deemed important by the program development team to reinforce participant education on healthy behaviors related to diet and exercise.

### Application to the process evaluation interview questions

Cataloging results from the beta-testing “think aloud” interviews provided a foundation for developing semi-structured interview questions to be used during the subsequent process evaluation of the AMPLIFY program. Each step in this process highlighted topical areas and potential questions to carry over from the beta-testing stage into the subsequent process evaluation. Interview questions were then developed based directly on the beta-testing results. Examples of these questions and the progression from beta-testing results to evaluation questions are provided in Table 2.

The process evaluation interview questions aimed at exploring a range of features within the AMPLIFY program but were deliberately broad to ensure the participant was able to discuss features they considered engaging, satisfying, motivating, or useful. Several refinements also highlighted the need to incorporate interview questions exploring overall perceptions of the program. For example, one participant noted difficulties in navigating the website and suggested incorporating a back arrow function to allow navigation between old and new program content: “you can get more information…but it won’t just back automatically. I would probably have a back arrow, going back.” Based on this suggestion, the program development team considered this a feasible refinement and incoporated a back arrow function into the website. The refinement was categorized as relating to satisfaction and usefulness. An example process evaluation interview question was developed to explore this concept; such as, “what features made navigating the website easy/difficult.”

## Discussion

We described an approach to use beta-testing results to inform individual interview questions for use during a process evaluation conducted during efficacy testing of a web-based diet and exercise program. The application of beta-testing results to program refinements and the development of process evaluation interview questions provides a potential guide for other researchers developing user-centered digital health program evaluations. While we strategically tailored the resultant process evaluation questions to the AMPLIFY program, the types of questions generated are consistent with other published process evaluations of web-based diet and exercise programs enhancing potential replicability of our results and applicability to other interventions [16,18,19,22,23]. For example, the interview questions generated focused on program motivation for healthy living (e.g., “how did you use the AMPLIFY program to help you reach your goals”) and program satisfaction (e.g., “how would you describe your satisfaction with the weight tracking tool”). Further, linking and aligning refinements to constructs identified in the systematic literature review reiterated the validity of including these constructs in future process evaluations [14,20,24,25]. Linking refinements to process evaluation constructs is a new contribution to the literature and may support other evaluators interested in user-centered evaluation designs.

Importantly, the variations in the four constructs (engagement, satisfaction, motivation, and usefulness) associated with each phase of the beta-testing “think aloud” interviews suggest that qualitative components of the process evaluation should occur at multiple timepoints during efficacy testing (Table 2). Similar to that reported by others, collecting qualitative data at multiple timepoints can enrich the evaluation and explore how participants’ perspectives may change throughout the program timeline [18,26].

While our results are consistent with several other process evaluations, it should be noted that process evaluations are frequently designed to reflect the program context and study aims. Thus, wide variations exist in the design of process evaluations and the number of constructs explored; such that, many process evaluations focus exclusively on supporting constructs without associating the supporting constructs to complex or broadly defined constructs [27–29]. For example, a process evaluation involving a home-based intervention for metabolic syndrome among adults living with chronic disease explored the construct of motivation without linking motivation to program use [27]. Another study explored the construct of satisfaction without suggesting satisfaction may be related to program acceptability [29]. In addition, multiple other constructs were identified in the context of our systematic review that were not considered relevant to the process evaluation of the AMPLIFY program [15]. However, it should be noted that our analyses were designed to reflect a specific study context, timeframe, and study aim; yielding further evidence supporting important evaluation constructs and a deeper understanding of refinements that may be needed to meet program improvement goals.

Several notable strengths exist for our approach and analyses. Our focus on developing a user-centered process evaluation methodology with potential to improve digital health acceptability and use (downstream effects of achieving engagement, satisfaction, motivation, and usefulness) among older cancer survivors is of high public health significance. The vast majority of the millions of cancer survivors living in the U.S. are over the age of 60 and do not meet diet, exercise, and/or weight management recommendations [30–32]. Hence, evidence-based process evaluations are needed to ensure scalable, digital health programs are acceptable and engaging to this high-need group. Further, our inclusion of 53% women and 41% minority beta-testers increases the potential generalizability of our findings to other studies that refine interventions and develop process evaluation interviews. Also, we clearly noted the progression across the analytical steps, illustrated by moving from beta-testing “think aloud” interview results, to identifying program improvement goals, and lastly to developing process evaluation interview questions (Table 2).

We also acknowledge several study limitations. Replication of our approach and an exploration of the benefits of this approach with a larger sample size, in different study contexts, and with other racial/ethnic groups is needed to explore the usefulness of this method in other settings. Some of the challenges we faced during the “think aloud” interview process also included ensuring that any comments or suggestions were clearly stated by the beta-tester so that we could adopt the most relevant and accurate change. Oftentimes, it is difficult for beta-testers to communicate the exact change they would like incorporated and can be difficult to offer suggestions for refinements in this setting. Further, the evaluation constructs (engagement, motivation, satisfaction, and usefulness) have overlapping and broad definitions which made it difficult, at times, to distinguish between each construct and application in an actual process evaluation. Clear distinctions are needed to better understand the exact role each construct maintains in a process evaluation [33].

## Conclusions

Process evaluations are increasingly common and can be utilized to evaluate constructs relevant to program acceptability and use from the perspective of participants and end-users [33–35]. Based on our findings, linking results from beta-testing “think aloud” interviews during program efficacy testing can inform user-centered process evaluation interview questions for use during subsequent study stages. Investigators conducting process evaluations during efficacy testing of web-based diet and exercise programs may consider the value of a similar approach to ensure a consistent focus on user-centered evaluations. We recommend additional research exploring variations in the link between beta-testing methods, hypotheses, and constructs that may further define the value of linking beta-testing results with process evaluations, including the potential role of artifical intelligience (e.g., large language models and generative articial intelligence) in improving generalizability and efficiency. However, it should be noted that there are numerous approaches to program beta-testing and linking results from one stage of web-based program development to another stage may not be practical or valuable in some instances. Thus, future research involving user-centered process evaluations may focus on establishing the value of these approaches and identifying the most appropriate contexts to utilize these methods.
